# Expectations and experiences of women regarding maternal healthcare services in Pakistan: challenges and lessons to be learnt

**DOI:** 10.1186/s40545-021-00392-x

**Published:** 2021-12-19

**Authors:** Madeeha Malik, Katherine Prescott, Maliha Khalid, Ayisha Hashmi, Ayyaz Kiani

**Affiliations:** 1 Hamdard Institute of Pharmaceutical Sciences, Hamdard University, Islamabad, Pakistan; 2 Private Company, Islamabad, Pakistan; 3 Cyntax Health Projects Pvt Ltd, Contract Research Organization, Islamabad, Pakistan

**Keywords:** Maternal health, Maternal care services, Pre-birth, Post-birth, Women, Healthcare, Pakistan

## Abstract

**Background:**

Access to maternal healthcare services is an essential pre-requisite for improving women’s health. However, due to poor access and underutilization, women in developing countries remain vulnerable to various complications. Evaluation of quality maternal healthcare services in any country must include the opinions of the women being as a key stakeholder utilizing maternal healthcare services.

**Aim:**

The present study was designed to evaluate the experiences, perceptions and expectations of pre-birth and post-birth women regarding utilization and delivery of maternal healthcare services in Pakistan.

**Methodology:**

A qualitative study design was used. Snow ball sampling technique was adopted to identify the respondents. Interviews were conducted using semi-structures interview guide till saturation point was achieved. The sample size at saturation point for different respondents was: pre-birth women (*n* = 9) and post-birth women (*n* = 9). All interviews were recorded after getting permission from the respondents. The interviews were transcribed verbatim and were then subjected to thematic analysis.

**Results:**

The age group for the pre-birth respondents was 23–43 years while for post-birth group it was 23–32 years. Most of the respondents from both groups were from urban setting. Most of them were either first time pregnant or were having experience of one pregnancy. Thematic analysis of the interviews yielded different themes and sub-themes including birth experience, maternal treatment pathway, identified barriers for quality maternal care, involvement in healthcare decision-making, impact of Covid 19, payment dynamics, role of digital health and recommendations for improving maternal care services.

**Conclusion:**

The results of the present study concluded that the overall quality of the maternal care services provided in Pakistan was not up to the mark. High rate of caesarian section was prevalent. Majority of the women were not involved in the decision-making process or provided with any birth plan or counselling regarding birth signs, family planning, danger and birth signs. The cost of maternal care was quite high and not affordable for all.

## Background

Access to integrated, evidence-based and cost-effective maternal health services for reducing maternal and child mortality have remained a priority goal in the healthcare systems throughout the world [1]. Delivery of antenatal, delivery and post-natal care services have been considered as one of the prime parameters for achieving third goal of Sustainable Development Goals (SDG), i.e., health and well-being. It has been projected that by the end of the year 2030, measures should be taken to reduce maternal mortality ratio by 70 per 1,00,000 live births [2]. In order to achieve this goal, various interventions have been carried out in different countries to improve access as well as utilization of provided maternal health services through awareness sessions, patient education, telemedicine services, digital mobile applications and opening of specialized mother and child healthcare centers [3]. Despite implementation of these strategies to strengthen maternal care delivery system, the maternal mortality rate is still rising in developing countries. It has been estimated that 800 women die from pregnancy-related preventable causes and during delivery, whereas, the global maternal mortality ratio has been estimated to be 211 deaths per 100,000 live births which is mostly due to post-partum hemorrhage, eclampsia and sepsis [4].

Access to maternal healthcare services is an essential pre-requisite for improving women’s health. However, due to poor access and underutilization, women in developing countries remain vulnerable to various complications [5]. Various factors have been attributed to inadequate utilization and delivery of maternal healthcare services in developing countries including poor health seeking behavior, low health literacy, poor antenatal care provided by prescriber, unattended delivery by healthcare professional, accessibility to healthcare settings, high cost of pre- and post-natal care in private healthcare facilities, poor counseling and dissatisfaction of patient [6]. The emergence of Covid-19 pandemic has further worsened the situation with restrictions in movement, anxiety of being prone to infection and fear of visiting healthcare facilities being prime reasons for underutilization of the maternal care services [7]. Furthermore, lack of provision of adequate care due to overburdened healthcare facilities in low-resource countries has resulted in increased maternal morbidity and mortality during Covid-19 pandemic. The healthcare professionals are unable to provide quality care due to strict precautions affecting the prescriber and mother interaction [8].

Maternal mortality ratio (MMR) in Pakistan has decreased from 276 deaths per 100,000 live births as per Pakistan Demographic and Health Survey of 2006–2007 to 186 per 100,000 live births in 2017, still being highest in the South Asian region. Approximately 65% of the pregnant women in Pakistan seek routine antenatal care, half of the deliveries occur in absence of a skilled maternal care provider and less than 50% seek post-partum healthcare services [9]. Despite the presence of Maternal and Child Health Integrated Program in the country, the health seeking behavior for maternal services is poor among women [10]. Evaluation of quality maternal healthcare services in any country must include the opinions of the women being as a key stakeholder utilizing maternal healthcare services. In order to design interventions to improve the access and utilization of such services, it is necessary to evaluate the opinions, experiences and expectations of key stakeholders which include clinicians as well as mothers [11]. Limited data are available regarding expectations and experiences of pregnant mothers and post-partum women in seeking maternal healthcare services in Pakistan. Therefore, the present study was designed to evaluate the experiences, perceptions and expectations of pre-birth and post-birth women regarding utilization and delivery of maternal healthcare services in Pakistan.

## Methods

### Study design

A qualitative study was designed to evaluate the experiences, perceptions and expectations of pre-birth and post-birth women regarding utilization and delivery of maternal healthcare services in Pakistan.

### Study site and respondents

Study site for this research included household, universities and healthcare facilities located in twin cities, i.e., Islamabad and Rawalpindi of Pakistan. Study respondents included pre-birth and post-birth women willing to participate in the study.

### Sample size and sampling technique

Snow ball sampling technique was adopted as it is the best way of identifying the respondents having common characteristics. Interviews were conducted till saturation point was achieved. The sample size at saturation point for different respondents was: pre-birth women (*n* = 9) and post-birth women (*n* = 9).

### Data collection tool

After extensive and critical literature review, a semi-structured interview guide was developed and used as a study tool. To get detailed views of respondents, the focus was to design questions as open as possible. The tool comprised different sections consisting questions along with probing questions regarding demographic characteristics, accessibility to services, information around health and well-being, payment dynamics and identified problems with maternal care and recommendations. Face and content validation of interview guide was done by panel of experts. Pilot testing was conducted to seek objective opinion regarding making questions easier to understand, avoid bias or leading questions and/or avoid any potential ambiguity.

### Data collection procedure and analysis

The selected respondents were contacted either personally or through phone for getting interview appointment date and time. Written consent was obtained from the study respondents before conducting interview. When necessary, probing questions were used. Each interview lasted approximately 20–30 min. Every respondent was given chance to express her views at the end of interview session. All interviews were recorded after getting permission from the respondents. The interviews were transcribed verbatim and were then subjected to thematic analysis using bottom-up approach.

## Results

### Demographic characteristics of the respondents

The age group for the pre-birth respondents was 23–43 years while for post-birth group it was 23–32 years. Most of the respondents (88%; *n* = 16) from both groups were from urban setting. Most of them (88%; *n* = 16) were either first time pregnant or were having experience of one pregnancy. Nearly half of the total respondents (*n* = 10, 52.6%) from both groups were working women. Furthermore, healthcare cost of majority of the total respondents (*n* = 13, 68.4%) was covered by medical insurance. C-section rate was seen quite high among the respondents and all of them were using smartphone (Table 1).

**Table 1:** Demographic characteristics of the respondents

		Pre-birth		Post-birth	
		*n*	%	*n*	%
Setting	Rural	2	22%	0	0%
	Urban	7	78%	9	100%
Education	Bachelors or lower	5	56%	3	33%
	Masters or higher	4	44%	6	67%
Employment	Employed	5	56%	5	56%
Insurance	Covered	5	56%	8	89%
Pregnancy status	First Preg	6	67%	6	67%
	Second Preg	2	22%	2	22%
	Third or higher	1	11%	1	11%
Mode of delivery	Normal	2	22%	7	78%
	C-section	1	11%	2	22%
	Unknown	6	67%	0	0%
Smartphone	Access	9	100%	9	100%

### Experience of post-birth women regarding healthcare services after delivery

The admission of almost all the respondents was in time except one. Majority had good experience regarding cleanliness of the facility, had privacy during their stay and skin-to-skin contact. Nearly half of the respondents reported initiation of early breast feeding after the delivery by the staff. Most of the respondents reported respectful behavior of the staff towards them and they were communicating with them during labor. However, majority of the respondents were not counseled regarding danger signs and family planning after delivery (Table 2).

**Table 2:** Experience of post-birth women regarding healthcare services after delivery

Code	Online admission	Cleanliness	Privacy	Skin-to-skin contact	Early initiation of breast feeding	Respectful behavior	Communication during labor	Counseling on danger signs	Family planning counseling
Pos-B01	Yes	Very clean	Yes	Yes	No	Yes	Yes	No	Yes
Pos-B02	Yes	Very clean	Yes	Yes	Yes	Yes	Yes	No	No
Pos-B03	Yes	Very clean	Yes	Yes	Yes	Yes	Yes	No	No
Pos-B04	Yes	Very clean	Yes	Yes	Yes	Yes	Yes	No	No
Pos-B05	Yes	Very clean	Yes	Yes	No	Yes	No	No	No
Pos-B06	Yes	Somewhat clean	No	Yes	No	Yes	No	No	No
Pos-B07	Yes	Very clean	Yes	Yes	Yes	Yes	Yes	Yes	Yes
Pos-B08	Yes	Very clean	Yes	Yes	No	Yes	Yes	No	No
Pos-B09	Slight delay	Very clean	Yes	Yes	No	Somewhat	No	No	No

### Theme 1: Perceptions regarding maternal care services

The pre-birth respondents had mixed experience regarding maternal care services irrespective of the healthcare facility they were visiting. Nearly half of them had high expectations based on their previous experience and were ready for the birth. On the other hand, most of the post-birth respondents did not have previously good experience and changed their doctors more frequently. All the respondents except one were not prepared for the birth.

- **Birth experience**
  *“I visited the same doctor initially for four months of pregnancy but my experience was not good as I became more ill and weaker by the treatment given. I switched my doctor and now my experience is going very good as it’s my ninth month”* ***(Pre-B 03)***
  *“My last experience has been very good so I am expecting the same level of service this time too”* ***(Pre-B 05)***
  *“I had difficulty in finding the doctor as I was very scared because of my previous miscarriage. However, I chose the doctor during my second pregnancy on recommendation of my aunt”* ***(Pos-B 02)***
  *“I had great difficulty in finding the right doctor. Then, I chose this doctor due to good feedback provided by my friends”* ***(Pos-B 05)***
- **Preparedness for birth**
  *“I feel myself ready for the birth as this is my third time and I do not have any stress or fear”* ***(Pre-B 01)***
  *“I am a bit confused at the moment and do not feel myself ready for the birth”* ***(Pre-B 02)***
  *“I was not prepared for the birth and was scared till the end”* ***(Pos-B 06)***
  *“As it was my second pregnancy so I felt ready as I knew all the process”* ***(Pos-B 09)***

### Theme 2: Maternal treatment pathway

Almost all the pre-birth as well the post-birth respondents stated that they were regularly called by their doctor every month for checkup. They were prescribed tests including blood CP, urine RE and ultrasound on every visit.

*“My expectations from the healthcare system is that I receive quality care. My doctor calls me monthly for checkup and prescribe me tests such as blood CP, urine RE on different visits”* ***(Pre-B 01)***

*“My expectations from the healthcare system are high in terms of quality and care from my doctor. I am called every month by my doctor up till now as this is my sixth month of pregnancy. She prescribes different tests namely blood CP, glucose, anomaly scan, hepatitis testing and ultrasound on every visit”* ***(Pre-B 04)***

*“My doctor used to call me every month on the same date. My blood pressure, blood glucose level and ultrasound were performed on every visit while complete blood count and hemoglobin were checked after every two months”* ***(Pos-B 01)***

*“My doctor used to call me every month for checkup for two trimesters, then I was called after 15 days during the last trimester. I went to one doctor during first trimester then changed my doctor as a friend suggested another one who was rated better than this one. Ultra sound was performed regularly, however, complete blood count was done after second trimester”* ***(Pos-B 03)***

### Theme 3: Identified barriers in provision of quality maternal care services

Nearly all the pre-birth as well as post-birth respondents stated that they faced difficulty in finding the right doctor. They usually chose doctor based on the recommendation of friends, family members, colleagues and/or internet reviews. Few of the pre-birth and post-birth respondents highlighted that they were not able to give proper feedback and the healthcare facility also lack follow-up. Almost all of the pre-birth and post-birth respondents were of the view that they were not involved in the decision-making process.

- **Finding a doctor**
  *“Yes, I had difficulty in searching the right doctor. Although, my family members recommended another doctor but I selected this doctor myself on the basis of reviews. There should be some sort of database of gynecologists which could be reviewed by the patients for in terms of ratings and reviews of previously treated patients as this can help to choose the right doctor”* ***(Pre-B 02)***
  *“It is a bit difficult to search for a good doctor specially during the Covid period as one cannot go freely to the hospitals and get information. I choose my doctor on recommendations of my family and friends”* ***(Pre-B 07)***
  *“I had difficulty in finding this doctor as I was very scared because of my previous miscarriage. However, during my second pregnancy I chose the doctor based on the recommendation of my aunt”* ***(Pos-B 02)***
  *“I had great difficulty in finding the right doctor. I chose this doctor based on the feedback of my friends. There should be an app for finding the doctor”* ***(Pos-B 05)***
- **Mechanism for feedback and follow-up**
  *“No, I am not able to give feedback. The hospital doesn’t have a very good feedback mechanism. Although, they have a complaint box in which one can drop their complaint and then they contact the complainant”* ***(Pre-B 01)***
  *“Yes, I am able to give feedback to my doctor. At times I feel very anxious regarding pregnancy complications or delivery and get panic but my doctor console me well”* ***(Pre-B 07)***
  *“No, I was not able to give feedback as doctor never listened to my complaints. Even I was guided wrong about estimated due date (EDD)”* ***(Pos-B 05)***
  *“Yes, I was able to give feedback. I was concerned regarding my gestational diabetes and got panic but she consoled me well”* ***(Pos-B 07)***
- **Informed decision-making**
  *“No, I haven't been able to make choices myself. My doctor makes the choices”* ***(Pre-B 02)***
  *“No, I don’t feel able to make informed choices”* ***(Pre-B 07)***
  *“I was not involved in decision-making at all”* ***(Pos-B 02)***
  *“No, I was not able to make informed choices neither involved in decision-making”* ***(Pos-B 03)***

### Theme 4: Choice in healthcare decision-making

Nearly all the pre-birth respondents reported that even if they have choice in healthcare decision-making still they follow their doctor’s recommendations for every decision or their family members makes decisions for them. On the other hand, all the post-birth respondents were not involved in decision-making or provided with any birth plan or counselling regarding birth signs.

*“My doctor guides me about the available choices. I follow her recommendations”* ***(Pre-B 08)***

*“I don’t have a say in my healthcare. I followed the doctor’s recommendations as it is. The doctor mainly makes the decision. Beside this, my husband makes the decisions for my healthcare”* ***(Pre-B 02)***

*“I was not involved in the decision-making at all. All the decisions were made by my doctor”* ***(Pos-B 08)***

*“No, I didn’t have a say in my healthcare. I followed the instructions of my doctor”* ***(Pos-B 09)***

### Theme 5: Source of knowledge regarding maternal care

Nearly all the pre-birth as well as the post-birth respondents highlighted that the most common source for gathering information used was via google followed by Facebook, WhatsApp group and/or their doctor.

*“I search queries related to pregnancy on the internet or mobile apps and also ask my doctor through WhatsApp”* ***(Pre-B 05)***

*“I search queries on google, Facebook and mobile apps and also ask my doctor through WhatsApp”* ***(Pre-B 09)***

*“I used to ask doctor on my appointment or use to call her. Moreover, I use to search internet for information but never used any helpline”* ***(Pos-B 01)***

*“Although, I used to ask my doctor *via* WhatsApp but still used to search google and YouTube for information but never used any helpline”* ***(Pos-B 04)***

### Theme 6: ProvisiZon of maternal care counselling

Almost all the pre-birth as well as the post-birth respondents indicated that they did not receive any counselling from their doctor due to her excessive workload. The respondents highlighted that counselling regarding family planning, danger signs and birth signs were not provided to them.

*“I have not been given any information on danger signs and family planning till now”* ***(Pre-B 01)***

*“No, I have not been counseled on warning signs and family planning”* ***(Pre-B 04)***

*“I did not receive any birth plan or was guided about birth risks or family planning”* ***(Pos-B 05)***

*“I did not receive any birth plan or was guided about birth risks”* *(****Pos-06)***

### Theme 7: Impact of COVID on maternal care

Nearly all the pre-birth respondents except one stated that they did not feel safe travelling to the hospital during the COVID 19 pandemic, despite the necessary SoP being followed at the hospitals. Contrary to this, mix responses of post-birth respondents were seen regarding safety concerns for travelling to hospitals during Covid 19.

*“I do not feel safe during this time. I use my own car but still the hospital environment makes me scared during Covid 19”* ***(Pre-B 05)***

*“Yes, I feel safe traveling to the hospital during Covid 19 as I use my own vehicle. However, my doctor has advised me not to visit in the first few months of pregnancy due to Covid 19 until and unless it is necessary”* ***(Pre-B 01)***

*“No, it didn’t feel safe to go to the hospital during Covid 19, so I preferred that the doctor guided me over the phone call”* ***(Pos-B 09)***

*“I found it safe to travel to hospital during Covid 19”* ***(Pos-B 08)***

### Theme 8: Payments dynamics for maternal care

All the pre-birth as well as post-birth respondents highlighted that the cost of maternal care was quite high and not affordable for all. Half of the pre-birth while all the post-birth respondents except one had their treatment covered through insurance. Moreover, all the pre-birth as well as post-birth respondents highlighted the need of reducing cost of care in order to make it easily affordable and accessible for all.

- **Cost and mode of payment**
  *“Healthcare cost is a burden for us as we do not have any medical insurance nor we have planned to have one yet”* ***(Pre-B 02)***
  *“Healthcare cost is quite high. My healthcare cost is fully paid by the insurance company at my workplace”* ***(Pre-B 08)***
  *“Healthcare cost is an immense burden especially in case of C section. My healthcare is paid by insurance company at my workplace”* ***(Pos-B 01)***
  *“It is a burden as the cost is very high for birth. We bear the cost ourselves. We do not have any intensions to go for insurance as the process is complicated”* *(****Pos-B 09)***
- ** Expected treatment cost**
  *“I expected it to be less than Rs. 60,000 but it is more than Rs. 80,000 for normal delivery and more than Rs.1,50,000 for a cesarean delivery”* ***(Pre-B 03)***
  *“I expected to pay less than Rs. 30 thousand by the end”* ***(Pre-B 09)***
  *“It should be free or less than Rs. 20,000 in order to be accessible and affordable for all”* ***(Pos-B 07)***
  *“It should be less than Rs. 50,000 to be affordable for everyone”* ***(Pos-B 04)***

### Theme 9: Role of digital health in improving maternal care

All the pre-birth as well as post-birth respondents acknowledged the role of digital health for improving maternal care. A mobile app was the most recommended option for improving maternal care followed by helpline/website and WhatsApp group by the pre-birth respondents while the post-birth women suggested class room training and telemedicine as the most effective digital health strategy for improving maternal care.

*“There should be a mobile app for asking queries and WhatsApp group could be effective”* ***(Pre-B 03)***

*“There should be a helpline and a WhatsApp group for mothers”* ***(Pre B 05)***

*“I think class training or WhatsApp group could be effective. Beside this, I think telemedicine could also be effective during this pandemic as counselling could be easily received at home”* ***(Pos-B 02)***

*“I think class training will be effective. Recorded videos might be played in the waiting areas. Moreover, I think telemedicine might also be effective to some extent keeping in view the literacy rate of Pakistan”* ***(Pos-B 05)***

### Theme 10: Recommendations for improving maternal care services

All the pre-birth as well as post-birth respondents suggested to improve the maternal care services by establishing a database of gynecologists, providing birth plan and appropriate guidance/counselling to the new mothers regarding danger signs, miscarriage, life style modification, lactation, post-partum depression, birth signs and complications associated with pregnancy as well as delivery.

*“There is a need of counseling and guidance sessions by the doctor regarding treatment. Doctors should be empathetic and should motivate the mother. New mothers should know about the specific diet and danger signs and symptoms. There should be a separate counseling service within the hospital for guidance regarding diet and other pregnancy related queries”* ***(Pre-B 01)***

*“New mothers should be counseled about signs and symptoms of each stage of pregnancy. Diet counseling should also be provided. Treatments should be clearly explained. Husbands should be involved in the whole process and also be guided by the doctors. The facilities should be accessible to patients”* ***(Pre-B 02)***

*“Patient education and counselling should be available at every stage of pregnancy. Patients should be counseled regarding self-care and lactation. Dietary and post-partum depression counselling must be provided”* ***(Pos-B 01)***

*“Unnecessary tests and visits for checkup should be avoided to reduce cost. Gynecologist fee should be affordable for everyone. New mother must be counseled regarding OTC medicines intake, life style and diet”* ***(Pos-B 04)***

## Discussion

During the past decade, a significant improvement has been observed in the quality and delivery of maternal healthcare services throughout the world by implementing specialized maternal child health programs focusing on nutrition, medical care, pre-partum and post-partum services. However, despite these interventions it has been recorded that more than half a million women die during pre-partum and post-partum phase, especially in the developing countries. The reasons might include poor access and reduced utilization of maternal care services along with various social, economic, and cultural factors contributing towards rising maternal morbidity and mortality in developing countries including Pakistan [1]. It is important to evaluate the experiences, perceptions and expectations of pre-birth and post-birth women regarding utilization and delivery of maternal healthcare services as being the key beneficiaries this might help in developing interventions which could be accessible and helpful in improving the quality of maternal healthcare services in the country. The results of the present study reported high rate of C-section among the selected respondents. Pre-birth respondents had mixed experience regarding maternal care services irrespective of the healthcare facility they were visiting. Nearly half of them had high expectations based on their previous experience and were ready for the birth. On the other hand, most of the post-birth respondents did not have previously good experience and changed their doctors more frequently. Moreover, all the respondents of both groups acknowledged the role of digital health for improving maternal care. Nearly all of them highlighted that the most common source for gathering information used by them was via google followed by Facebook, WhatsApp group and/or their doctor. However, a mobile app was the most recommended option for improving maternal care followed by WhatsApp group and helpline/website by them. Furthermore, classroom-training and telemedicine were also seen as effective strategies for improving maternal care by post-birth women. A study conducted in Australia reported that women were adopting digital technologies leading to increased use of mobile health applications for pregnancy care [14]. It is necessary that prescriber's provide adequate information regarding birth choices as well as involve women in shared decision-making regarding management of the condition in order to increase their satisfaction with the provided services [12]. The results of the present study reported that nearly all the pre-birth respondents reported that even if they have choice in healthcare decision-making still they follow their doctor’s recommendations for every decision. On the other hand, all the post-birth respondents were not involved in decision-making or provided with any birth plan or counselling regarding birth signs. A study conducted in Canada reported similar findings that mothers received inadequate information during their pre-natal care by their prescriber’s [13].

Counseling can help improve the utilization of healthcare services as well as might reduce maternal complications [7]. The results of the present study reported that all the pre-birth as well as post-birth respondents suggested to improve the maternal care services by establishing a database of gynecologists, providing birth plan and appropriate guidance/counselling to the new mothers regarding danger signs, miscarriage, life style modification, lactation, post-partum depression, birth signs and complications associated with pregnancy as well as delivery. Similar findings were reported from a study conducted in Iran where individualized counseling of mothers led to improved health services utilization as well as initiation of breast feeding [17].

The emergence of Covid-19 pandemic has severely impacted the quality of healthcare services throughout the world. As pregnant women require specialized care, the delivery of such services due to pandemic has been negatively affected [7]. The results of the present study reported that nearly all the pre-birth respondents except one stated that they did not feel safe travelling to the hospital during the COVID 19 pandemic, despite the necessary SoP being followed at the hospitals. Contrary to this, mixed responses of post-birth respondents were seen regarding safety concerns for travelling to hospitals during Covid 19. Similar results were observed in a study conducted in UK where the frequency of prenatal visits reduced due to Covid-19 pandemic [7].

Access to quality evidence-based maternal care is necessary to reduce maternal mortality and morbidity. However, the cost of delivery and post-natal care can affect the utilization of these services [15]. The results of the present study reported that all the pre-birth as well as post-birth respondents highlighted that the cost of maternal care was quite high and not affordable for all. Half of the pre-birth while all the post-birth respondents except one had their treatment covered through insurance. Moreover, all the pre-birth as well as post-birth respondents highlighted the need of reducing cost of care in order to make it easily affordable and accessible for all. Similar results were obtained in a study conducted in Afghanistan where an increase in fee resulted in reduced visits to maternal clinics [16].

## Study limitations

As in any study, it is only possible to reflect the opinions and experiences of women who agree to participate. It is possible that the views of women who did not take part in this interview study may have differed from the views of those who did. Majority of the respondents were literate, from urban setting and moderate-income background, therefore, the experience and perceptions of the illiterate women or from rural areas and low social economic background might be different and were not captured by the study.

## Conclusion

The results of the present study concluded that the overall quality of the maternal care services provided in Pakistan was not up to the mark. High rate of caesarian section was prevalent. Majority of the women faced difficulty in finding the right doctor and were not prepared for the birth. They were not involved in the decision-making process or provided with any birth plan or counselling regarding birth signs, family planning and danger signs. The cost of maternal care was quite high and not affordable for all. The role of digital health for improving maternal care was acknowledged by the women. It was suggested by the respondents to improve the maternal care services by establishing a database of gynecologists, providing birth plan and appropriate guidance/counselling to the new mothers regarding danger signs, miscarriage, life style modification, lactation, post-partum depression, birth signs and complications associated with pregnancy as well as delivery. Thus, adequate support system should be in place at healthcare facilities to provide prenatal and post-natal support to all mothers to reduce maternal complications and promote early initiation of breastfeeding in Pakistan.

## Data Availability

All data are available and can be provided by the corresponding author upon rational request.

## Appendix

See Table 3.

**Table 3:** Detailed demographic characteristics of the respondents

Code	Code description	Age (years)	Setting	No. of pregnancies	Qualification	Employment status	Insurance	Mode of delivery	Smartphone
Pre-B01	Pre-birth	32	Urban	3rd	Bachelors	Employed	Insured	Normal	Yes
Pre-B02	Pre-birth	30	Urban	1st	Masters	Housewife	Not insured	–	Yes
Pre-B03	Pre-birth	23	Urban	1st	Bachelors	Housewife	Not insured	–	Yes
Pre-B04	Pre-birth	25	Rural	1st	Intermediate	Housewife	Not insured	–	Yes
Pre-B05	Pre-birth	27	Urban	2nd	Masters	Employed	Insured	C-Section	Yes
Pre-B06	Pre-birth	25	Rural	1st	Matric	Housewife	Not insured	–	Yes
Pre-B07	Pre-birth	34	Urban	1st	Masters	Employed	Insured	–	Yes
Pre-B08	Pre-birth	43	Urban	1st	Masters	Employed	Insured	–	Yes
Pre-B09	Pre-birth	35	Urban	2nd	Bachelors	Employed	Insured	C-Section	Yes
Pos-B01	Post-birth	29	Urban	2nd	Masters	Employed	Insured	C-Section	Yes
Pos-B02	Post-birth	30	Urban	3rd	Masters	Housewife	Insured	C-Section	Yes
Pos-B03	Post-birth	26	Urban	1st	Bachelors	Housewife	Insured	C-Section	Yes
Pos-B04	Post-birth	30	Urban	1st	Masters	Employed	Insured	Normal	Yes
Pos-B05	Post-birth	30	Urban	1st	Masters	Employed	Insured	C-Section	Yes
Pos-B06	Post-birth	26	Urban	1st	Masters	Employed	Insured	C-Section	Yes
Pos-B07	Post-birth	31	Urban	1st	Bachelors	Employed	Insured	C-Section	Yes
Pos-B08	Post-birth	23	Urban	1st	Bachelors	Housewife	Insured	Normal	Yes
Pos-B09	Post-birth	32	Urban	2nd	Masters	Housewife	Not insured	C-Section	Yes

## Abbreviations

Blood CP: Blood complete picture
*C-section*: Caesarian section
EDD: Estimated due date
Pre-B: Pre-birth
Pos-B: Post-birth
SDG: Sustainable Development Goals
UrineRE: Urine routine examination

## References

[1] Nuamah GB (2019). Access and utilization of maternal healthcare in a rural district in the forest belt of Ghana. BMC Pregnancy Childbirth.

[2] Kifle D (2017). Maternal health care service seeking behaviors and associated factors among women in rural Haramaya District, Eastern Ethiopia: a triangulated community-based cross-sectional study. Reprod Health.

[3] Rosário EVN (2019). Determinants of maternal health care and birth outcome in the Dande Health and Demographic Surveillance System area, Angola. PLoS ONE.

[4] Sitaula S, et al. Prevalence and risk factors for maternal mortality at a Tertiary Care Centre in Eastern Nepal-retrospective cross sectional study. 2021. 10.1186/s12884-021-03920-4 PMC8247237 34210273

[5] Haruna U, Dandeebo G, Galaa SZ (2019). Improving access and utilization of maternal healthcare services through focused antenatal care in rural Ghana: a qualitative study. Adv Public Health.

[6] Zelalem Ayele D (2014). Factors affecting utilization of maternal health care services in Kombolcha District, eastern Hararghe zone, Oromia regional state, eastern Ethiopia. Int Sch Res Not.

[7] Kotlar B (2021). The impact of the COVID-19 pandemic on maternal and perinatal health: a scoping review. Reprod Health.

[8] Temesgen K (2021). Maternal health care services utilization amidstCOVID-19 pandemic in West Shoa zone, central Ethiopia. PLoS ONE.

[9] Zain S (2020). The design and delivery of maternal health interventions in Pakistan: a scoping review. Healthc Women Int.

[10] Mumtaz Z (2012). Addressing disparities in maternal health care in Pakistan: gender, class and exclusion. BMC Pregnancy Childbirth.

[11] Bhutta ZA, Hafeez A (2015). What can Pakistan do to address maternal and child health over the next decade?. Health Res Policy Syst.

[12] Chen S-W (2018). Women’s decision-making processes and the influences on their mode of birth following a previous caesarean section in Taiwan: a qualitative study. BMC Pregnancy Childbirth.

[13] Debessai Y (2016). Inadequate prenatal care use among Canadian mothers: findings from the maternity experiences survey. J Perinatol.

[14] Lupton D (2016). The use and value of digital media for information about pregnancy and early motherhood: a focus group study. BMC Pregnancy Childbirth.

[15] Namasivayam V (2021). Association of prenatal counselling and immediate postnatal support with early initiation of breastfeeding in Uttar Pradesh, India. Int Breastfeed J.

[16] Dzakpasu S, Powell-Jackson T, Campbell OM (2014). Impact of user fees on maternal health service utilization and related health outcomes: a systematic review. Health Policy Plan.

[17] Shafaei FS, Mirghafourvand M, Havizari S (2020). The effect of prenatal counseling on breastfeeding self-efficacy and frequency of breastfeeding problems in mothers with previous unsuccessful breastfeeding: a randomized controlled clinical trial. BMC Womens Health.

